# The Roles of DNA Methyltransferases 1 (DNMT1) in Regulating Sexual Dimorphism in the Cotton Mealybug, *Phenacoccus solenopsis*

**DOI:** 10.3390/insects11020121

**Published:** 2020-02-12

**Authors:** Mohamed A.A. Omar, Meizhen Li, Feiling Liu, Kang He, Muhammad Qasim, Huamei Xiao, Mingxing Jiang, Fei Li

**Affiliations:** 1 Ministry of Agriculture Key Lab of Molecular Biology of Crop Pathogens and Insects/Institute of Insect Science, Zhejiang University, 866 Yuhangtang Road, Hangzhou 310058, Chinalimeizhen@zju.edu.cn (M.L.); lfeiling@zju.edu.cn (F.L.); hekang@zju.edu.cn (K.H.); cmqasimgill@zju.edu.cn (M.Q.); xiaohuamei625@163.com (H.X.); mxjiang@zju.edu.cn (M.J.); 2 Department of Plant Protection, Faculty of Agriculture (Saba Basha), Alexandria University, Alexandria 21531, Egypt; 3 College of Life Sciences and Resource Environment/Key Laboratory of Crop Growth and Development Regulation, Yichun University, Jiangxi Province, Yichun 336000, China

**Keywords:** Hemiptera, cotton mealybug, sexual dimorphism, DNA methylation, DNMT, RNAi

## Abstract

The cotton mealybug, *Phenacoccus solenopsis*, is an invasive pest that can cause massive damage to many host plants of agricultural importance. *P. solenopsis* is highly polyphagous, and shows extreme sexual dimorphism between males and females. The functions of DNA methyltransferase (DNMT) enzymes in the cotton mealybug have not been well studied. Here, we carried out an investigation of DNMTs in cotton mealybug to study their roles in sexual dimorphism. We found that the cotton mealybug has two copies of *PsDnmt1*, but *Dnmt3* is absent. We then amplified the full-length cDNAs of *PsDnmt1A* (2225 bp) and *PsDnmt1B* (2862 bp) using rapid amplification cDNA ends (RACE). Quantitative reverse transcriptase PCR shows that both *PsDnmt1A* and *PsDnmt1B* are highly expressed in adult males, while the expression of *PsDnmt1B* is 30-fold higher in gravid females than in virgin females. We knocked down *PsDnmt1A* and *PsDnmt1B* with small interfering RNAs (siRNAs), and both genes were successfully down-regulated after 24 h or 72 h in adult females and pupa (*t*-test, *p* < 0.05). Down-regulating the expression of these two *DNMT* genes led to offspring lethality and abnormal body color in adult females. Furthermore, the silencing of *PsDnmt1B* induced abnormal wing development in emerged adult males. Our results provide evidence that *PsDnmt1* plays a crucial role in regulating sexual dimorphism in the cotton mealybug.

## 1. Introduction

DNA methyltransferases (DNMTs) are a conserved family of enzymes that catalyze the methylation of cytosines in DNA, primarily in CpG dinucleotides. DNMTs play essential roles in regulating gene expression, chromosome stability, cell differentiation, development, and genomic imprinting [1,2,3,4,5,6]. DNMTs are divided into three types that differ in function: DNMT1 is responsible for maintenance of DNA methylation, and all DNMT1 paralogs might also be responsible for differentially maintaining DNA methylation temporally or spatially by methylating hemimethylated DNA regions [7]. However, the function of DNMT1 in DNA maintenance is still poorly understood, and further studies are required to explore the roles of these duplicated genes [8,9,10]. The second type of DNMT is DNMT2, which is involved in tRNA methylation, not DNA methylation. The last type is the DNMT3 family, which mainly works de novo to methylate new CpG sites and to modify DNA in response to environmental effects [9].

The pattern of DNA methylation is highly changeable during the development of insects [9,10,11]. Some insects lack DNMT3, whereas others have two copies of DNMT1 as a result of gene duplication. In these insects, the presence of DNA methylation mostly relies on maintenance rather than de novo DNA methyltransferases [9]. In some other insect species, three types of DNMTs are present; examples are the wasp *Nasonia vitripennis* and the honeybee *Apis mellifera* in the Hymenoptera, and the brown planthopper *Nilaparvata lugens* and the aphid *Acyrthosiphon pisum* in the Hemiptera [10,12,13,14]. However, some Dipteran species, such as the fruit fly *Drosophila melanogaster* and the mosquito *Anopheles gambiae*, have only DNMT2 [15,16]. In contrast, the silkworm *Bombyx mori* and the red flour beetle *Tribolium castaneum* both lack DNMT3 [13,17]. DNA methylation levels vary within insect species and, in some cases, do not rely on the presence of DNMT3. Surprisingly, some lepidopteran and blattodean species show a higher level of DNA methylation than do vertebrates and plants [9,18,19,20,21]. DNA methylation level may play an essential role in genomic imprinting and sex determination in mealybug [22]. In *Pseudococcus obscurus* and *Pseudococcus calceolariae*, the level of cytosine methylation is sex-specific, where it is higher in males than in females as well as being correlated with males heterochromatinization in both species of mealybugs [23].

The expression of *DNMT1* and *DNMT3* have been knocked down in many insect species, demonstrating that these genes play vital regulatory roles in insects. In the brown planthopper *N. lugens*, silencing *DNMT3* led to fewer offspring [10]. In the red flour beetle *T. castaneum*, silencing *DNMT1* led to early developmental arrest [24]. 

The cotton mealybug *Phenacoccus solenopsis* Tinsley (Hemiptera: Pseudococcidae) is a sap-sucking insect pest that causes significant plant damage to more than 166 plant species belonging to 51 different botanical families [25,26,27]. The adult males and adult females of this species are entirely different in appearance and show extreme sexual dimorphism: Adult females are larger in size, with a wingless body covered with wax, and they have three nymphal stages that are larva-like, whereas adult males are not covered with wax, are small in size with a pair of wings, and have two non-feeding developmental stages (prepupa and pupa) [28,29,30]. Male and female mealybugs have been reported to have different levels of DNA methylation [23]. However, the DNMTs have not been studied previously in the cotton mealybug, and their functions remain elusive. Here, we identified two copies of *DNMT1*, *PsDnmt1A*, and *PsDnmt1B*, in the cotton mealybug. We then amplified the full-length transcripts of *PsDnmt1A* and *PsDnmt1B,* examined their expression patterns, and knocked down these two genes in adult gravid females and the pupae of adult males. The results showed that *PsDnmt1A* and *PsDnmt1B* play crucial roles in regulating sexual dimorphism in *P. solenopsis*.

## 2. Materials and Methods 

### 2.1. Insects

Female cotton mealybugs (*P. solenopsis* Tinsley) were obtained from a cotton field at the Institute of Insect Sciences, Zhejiang University, Hangzhou city, Zhejiang province, China and maintained in the laboratory for 4 years without exposure to any insecticides. The mealybugs were reared on sprouted potatoes in plastic boxes in controlled climate chambers according to our previously described methods [29]. We collected at least three biological replicates with an estimated weight of ~500 ng/sample for each developmental stage, and all samples were flash-frozen in liquid nitrogen and stored at −80 °C prior to RNA extraction and use in the experiments.

### 2.2. Gene Identification and Bioinformatics Analysis

We performed a comprehensive bioinformatics search to determine whether genes of DNA methylation are present in the genome of the cotton mealybug. First, we downloaded the genome sequence of *P. solenopsis* from NCBI [31] (GenBank Accession: VFXL00000000). Then, six RNA-seq data (downloaded from NCBI, Accession: SRR7657827, SRR7657828, SRR7657829, SRR7657830, SRR7657831, SRR7657832 [29]), were aligned to the genome, generating a gene set with the HISAT-StringTie pipeline [32]. The gene sets were blasted to invertebrate proteins using NCBI/BLASTX (v2.7.1, E-value ≤ 1e−5). According to BLAST gene annotation, we obtained two copies of DNMT1 and one copy of DNMT2 candidates, namely *PsDnmt1A*, *PsDnmt1B*, and *PsDnmt2*. To get a complete set of DNMT1 candidate genes, we downloaded protein sequences of 1474 DNMT genes from NCBI (Species: animal; Source databases: RefSeq and UniProtKB/SwissProt) and used TBLASTN (v2.7.1) to search against the whole genome assembly. If the protein query had an E-value < 1e−5 and overall alignment coverage >30%, it would be considered as a DNMT candidate gene for further verification. Using this method, we did not find any other DNMT candidate genes. After we confirmed the presence of the most critical DNA methylation genes, we validated these genes using PCR amplification.

### 2.3. RNA Isolation and the Quantitative Real-Time Polymerase Chain Reaction (qRT-PCR) Analysis 

RNA was extracted from different developmental stages from first instar nymphs to adults with three biological replicates for each stage. Total RNA was extracted using a Trizol Total RNA Isolation user guide (Invitrogen, Life Technologies Corporation, Carlsbad, CA 92008 USA). Each RNA sample was dissolved in 30 μL of RNase-free water and then quantified using the UV5Nano spectrophotometer (Mettler Toledo; Thermo Fisher Scientific, Waltham, MA, USA), following the manufacturer’s protocol. Complementary DNA (cDNA) was synthesized from the RNA samples with a first-strand cDNA synthesis kit (Takara, Japan, Cat. 6110A) using 1 μg total RNA as the template, oligo (dT) primers, and RNase-free water in a 20 μL reaction following the manufacturer’s protocol. The *PsDnmt1A*- and *PsDnmt1B*-specific mRNA levels for all development stages were quantified using qRT-PCR assays with specific primers (Table 1). We used the *β-Actin* gene as a reference control for data normalization, and qRT-PCR was performed using an ABI 7500 Real-Time PCR System (Applied Biosystems, Foster City, CA, USA). We used the ChamQ SYBR^®^ qPCR color Master Mix (The Vazyme-China) with a standard qRT-PCR amplification protocol: pre-denaturation at 95 °C for 30 s, followed by 45 cycles of 95 °C for 10 s and 60 °C for 32 s. Melting curves were conducted by raising the sample temperature to 95 °C for 15 s, then cooling the samples to 60 °C for 60 s and heating again to 95 °C for 15 s. For each sample, there were three biological replicates, and each cDNA sample was repeated three times as a technical replicate. The relative changes in expression of the mRNA for each *PsDnmt1A* and *PsDnmt1B* gene were calculated with the 2^−ΔΔCT^ method [33].

### 2.4. Rapid Amplification of cDNA Ends (RACE)

PCR was used to amplify the fragments of *PsDnmt1A* and *PsDnmt1B*. We then used the RACE technique to obtain the full-length cDNAs of both genes with the SMART™ RACE cDNA Amplification Kit (Clontech, Mountain View, CA, USA). The 3′-UTR and 5′-UTR RACE cDNAs were synthesized from total RNA extracted as described in Section 2.3 using SMARTScribe™ Reverse Transcriptase (Clontech), according to the manufacturer’s instructions. We designed specific primers for *PsDnmt1A* and *1B* that were compatible with the universal primer in the RACE kit using PRIMER PREMIER 5.0 (Table 1). In the first PCR step, we used the gene-specific primers, and the Universal Primer (UPM) supplied in the kit. To obtain the 3′-UTR, we used one forward gene-specific primer with the UPM, and the 5′-UTR amplification used one reverse gene-specific primer with the UPM. The PCR conditions were as follows: incubation at 94 °C for 3 min; five cycles of 94 °C for 30 s and 72 °C for 3 min; five cycles of 94 °C for 30 s and 70 °C for 30 s, 72 °C for 3 min; and 25 cycles of 94 °C for 30 s, 68 °C for 30 s, and 72 °C for 3 min. There was a final extension step at 72 °C for 10 min. The PCR products were then separated by agarose gel electrophoresis, and DNA fragments of the predicted lengths were excised from the gel. After purification using a DNA gel extraction kit (Takara Mini BEST Agarose Gel DNA Extraction Kit Ver.4.0), the DNA fragments were cloned into the pMD™19-T Vector (Takara). The positive (white) colonies were selected, and the plasmids were extracted for DNA sequencing (Tsingke Biological Technology, Beijing, China).

### 2.5. Gene Phylogeny

The insect protein sequences from species in the different orders Hymenoptera, Lepidoptera, Hemiptera, Thysanoptera, Blattodea, Isoptera, Coleoptera, and Diptera were downloaded from NCBI [31], and saved in FASTA format. All accession numbers are given in **(**Table S2). We generated multiple sequence alignments of 103 DNMT amino acid sequences from eight insect orders using MUSCLE incorporated in MEGA X 10.1 (BETA) [34]. The evolutionary relationships were inferred by constructing phylogenetic trees using the Maximum Likelihood method and Poisson correction model [35]. The first tree for the heuristic search was done automatically using the Neighbor-Joining method, and the JTT model was used to estimate the BioNJ algorithms for a matrix of pairwise distances and to then select the topology with a superior log Maximum likelihood value. The evolutionary tree was drawn to scale, with branch lengths measured as the number of substitutions per site. The evolutionary tree was constructed in MEGA X 10.1 (BETA) [34]. 

### 2.6. RNA Inference

Three target siRNA sites for each gene were screened and synthesized by GenePharma Co., Ltd. [36]. along with negative control (Table 1). The small interfering RNAs (one OD_260_ with average molecular weight 13,300 g/mol) were dissolved in 125 μL diethylpyrocarbonate (DEPC-treated water) to a final concentration of 0.5 μg/μL. The micro-injection needles were pulled using a micropipette puller (Model P87, Sutter Instruments Co., Novato, CA, USA) from glass capillaries with a 1-mm outer diameter and 0.35-mm inner diameter. To avoid siRNA leakage from the adult female body, we kept the needles in the injection sites for 30 s. The siRNA injection experiment was performed using the Eppendorf InjectMan NI 2 microinjection system (Eppendorf, Hamburg, Germany). In each treatment, 30 adult gravid females were pooled into a single biological replicate and injected with 0.5 μg siRNA each. Approximately 30 pupae in each biological replicate were treated using the topical RNAi-based siRNA delivery method in which a few drops were applied to the dorsal side of the pupa abdomen at a final concentration of ~500 ng/μL following the methods described by [37]. The control groups underwent the same procedures under the same conditions, and all experiments were performed in triplicate. The time point for siRNA treatment was chosen based on the gene expression profile determined by qRT-PCR.

### 2.7. Statistical Analysis

We used SPSS 24.0 software (IBM, Chicago, IL, USA) for statistical analysis. The data are presented as means ± standard error (SE) of three biological replicates. The significant differences between more than three groups were calculated using a one-way analysis of variance (ANOVA) in conjunction with TUKEY test, and different litters are used to indicate significance at *p* <  0.05, while we used Student *t*-test (*p* < 0.05) to analyze the two groups. 

## 3. Results

### 3.1. The Cotton Mealybug Genome Encodes Two Copies of DNMT1 but Not DNMT3

**Figure 1:**
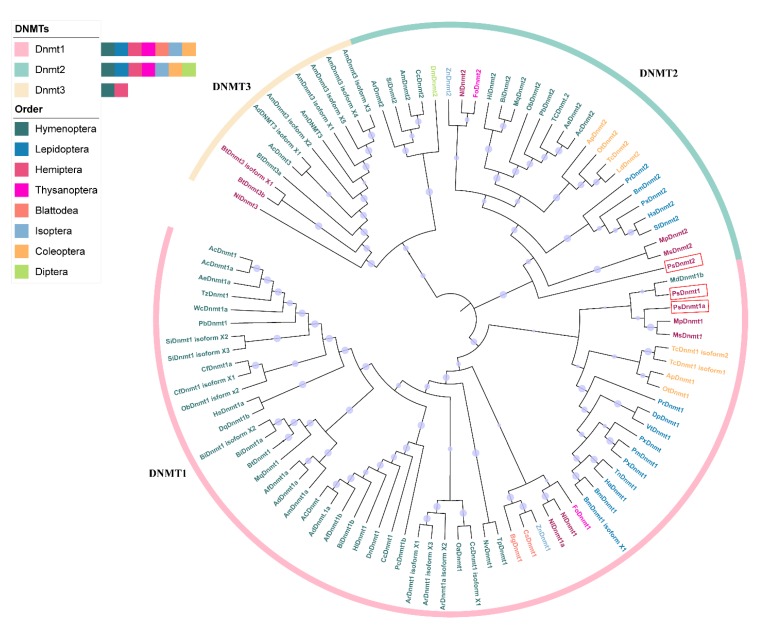
Phylogenetic analysis of the DNMT protein families (1, 2, and 3) across different insect orders. One-hundred-and-three amino acid sequences from eight insect orders were used to construct this tree using the Maximum Likelihood method. Different colored parts of the circle represent the different DNMT types, and the different insect orders are also color-coded. The sequences of proteins from the cotton mealybug, *P. solenopsis*, are enclosed in red boxes.

Two different copies of *DNMT1* and one copy of *DNMT2* were found, whereas *DNMT3* was found to be absent from the cotton mealybug genome. Since *DNMT2* is involved in tRNA methylation, not DNA methylation, we focused on the *DNMT1* for further study. We named these two copies of *DNMT1* as *PsDnmt1A* and *PsDnmt1B* based on the sequence similarities with other orthologous genes in insects (Figure 1). We validated these two genes with PCR using gene-specific primers (Table 1). Rapid amplification of cDNA ends (RACE) was then used to amplify the full-length transcripts of both genes, and we found that *PsDnmt1B* was longer than *PsDnmt1A*. The full-length cDNA of *PsDnmt1A* is 2225 bp, and consist of 230 bp of 5′-UTR, and 101 bp of 3′-UTR, encodes a predicted protein of 630 amino acids. Whereas *PsDnmt1B* cDNA is 2862 bp in length, comprising 392 of 3′-UTR and encodes a predicted protein of 822 aa (Table S1 and, Figure S1). The full-length cDNA sequences of *PsDnmt1A* and *PsDnmt1B* were submitted to GenBank under accession numbers MN696786 and MN696787, respectively (Table S1).

### 3.2. The Cotton Mealybug PsDNMT1 Proteins Possess Conserved Domain Structures

Domain analysis showed that both of the predicted PsDNMT1 proteins possess the main conserved domains of DNMTs, but the domain lengths and arrangement are different in the *P. solenopsis* proteins (Figure S1). PsDNMT1A consists of the catalytic domain (DNMT1-RFD), the cytosine-specific DNA methyltransferase replication foci domain, and the bromo-adjacent homology (BAH) domain. The BAH domain is likely to function as a protein–protein interaction module, especially in gene-regulation and silencing, and therefore plays a vital role by linking DNA methylation with replication and transcriptional regulation [38]. The PsDNMT1B protein contains another unique conserved domain of the DNMT family, the DNA-methylase domain, which is considered to be the central catalytic domain for its enzymatic activity, along with a pair of BAH domains (Figure S1).

### 3.3. PsDNMT1A and PsDNMT1B Cluster into Two Different Phylogeny Sub-Clades

We constructed a phylogenetic tree based on an alignment of 103 DNMT amino acid sequences from eight insect orders (Figure 1). All DNMT protein sequences were divided into three groups: maintenance DNA methylation genes (DNMT1), tRNA methylation genes (DNMT2), and de novo DNA methylation genes (DNMT3). The PsDNMT2 protein clustered with all other DNMT2 proteins from different insect orders. The DNMT3 cluster does not include a member from the cotton mealybug. Interestingly, PsDNMT1A clustered with MpDNMT1, which is from the green peach aphid (*Myzus persicae*), a hemipteran species. In contrast, PsDNMT1B clustered with MdDNMT1B from the hymenopteran parasitoid wasp *Microplitis demolitor* (Figure 1), suggesting that the two paralogous copies of DNMT1 in *P. solenopsis* have evolved different functions.

### 3.4. PsDnmt1A Is Highly Expressed in Males, While Psdnmt1b Is Abundant in Females

**Figure 2:**
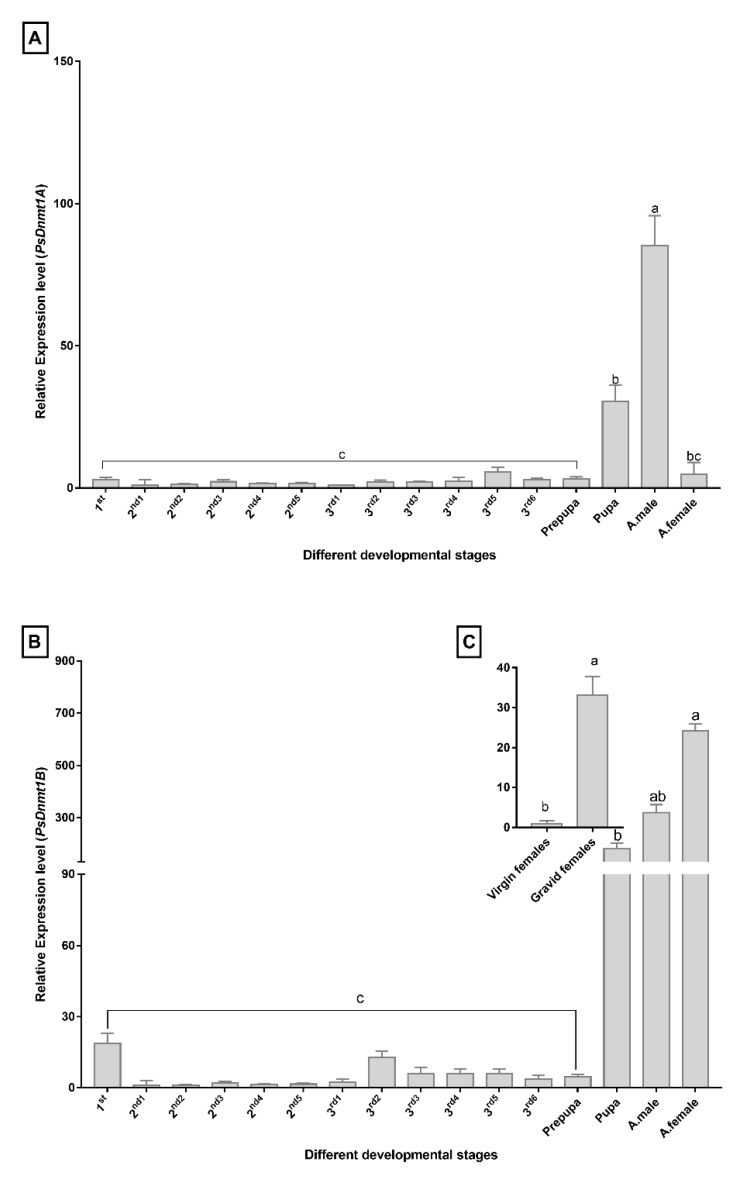
Expression profiles of *PsDnmt1A* and *1B* in all the different developmental stages of *P. solenopsis* at different times. (**A**) Expression levels of PsDnmt1A. (**B**) Expression levels of PsDnmt1B, the first day of the second instar nymphs (2^nd1^) were arbitrarily assigned a value of 1. (**C**) Expression of *PsDnmt1B* in virgin females and gravid females; virgin females were arbitrarily assigned a value of 1. Relative expression levels were normalized against the expression of *β-actin* and visualized as mean ± SE with three biological replicates. Values were analyzed by one-way ANOVA in conjunction with TUKEY test, while Student t-test was used to analyze two groups; and bars with different lowercase letters (abc) are used to indicate significance at *p* < 0.05.

We used RT-qPCR to assay the expression profiles of *PsDnmt1A* and *PsDnmt1B* during the entire life cycle of the cotton mealybug, including the first, second, and third nymphal stages, and the prepupa, pupa, adult male, and adult female. There were no significant differences in mRNA expression levels of *PsDnmt1A* or *PsDnmt1B* in any of the immature or early development stages (one-way ANOVA, *p* > 0.05, Figure 2A,B). However, there was a dramatic difference in the expression profile of *PsDnmt1A* between adult males and females of the cotton mealybug: *PsDnmt1A* showed significant expression levels in the male-specific stages (pupa and adult males) compared with the other stages (Figure 2A). *PsDnmt1B* exhibited a distinct expression pattern in which expression is highly upregulated in the first instar nymph (18-fold), and then sharply declines during the second and third instar nymphs until the pre-pupal stages. A significant difference in expression was observed in the pupa (180-fold) and adult males (319-fold), suggesting that *PsDnmt1B* has a more important role in regulating the development of male adults (Figure 2B). Also, *PsDnmt1B* showed significant expression levels in adult females (*p* < 0.001, Figure 2A). The relative abundance of *PsDnmt1B*-specific mRNA was higher in gravid females than in virgin females (*t*-test, *p* < 0.05, Figure 2C), indicating that the two *PsDnmt1* paralogs have different functions in regulating sexual dimorphism in the cotton mealybug.

### 3.5. PsDnmt1B Regulates Wing Development in Adult Males, While Psdnmt1a and 1b Modulate Adult Female Reproduction

**Figure 3:**
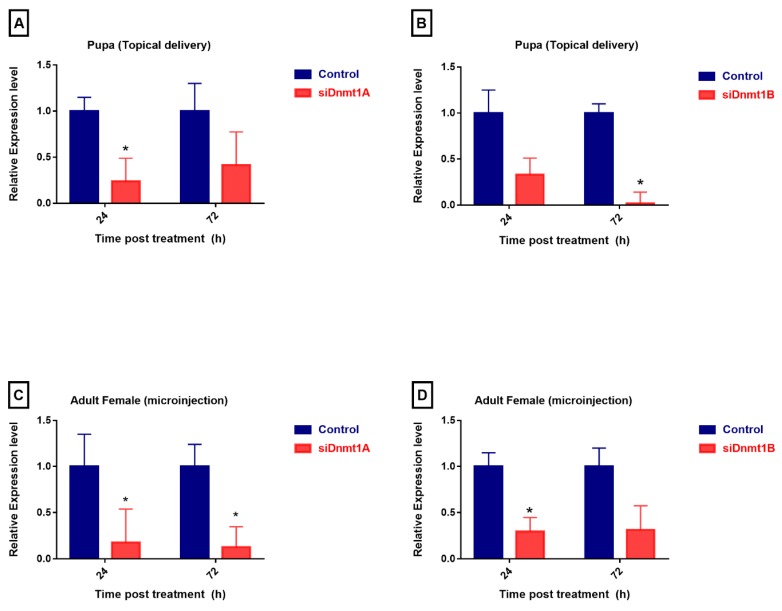
Relative mRNA levels of the two *DNMT* genes in pupae and adult females of *P. solenopsis* following topical delivery and microinjection with small interfering RNAs (siRNAs). At 24 h and 72 h post-treatment, the mRNA levels of *PsDnmt1A* and *PsDnmt1B* were significantly decreased compared with the control. Relative expression was normalized as mean ± SE with three biological replicates and analyzed using Student’s *t*-test (* *p* < 0.05).

**Figure 4:**
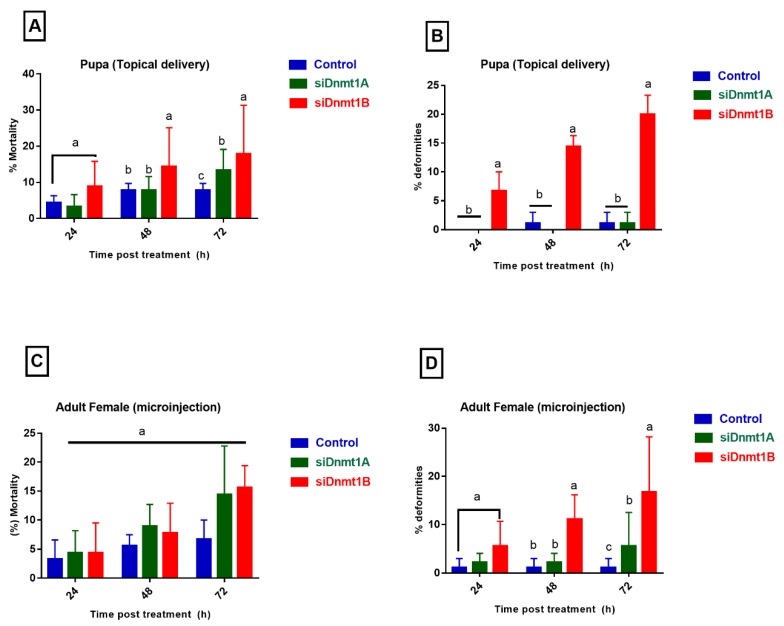
Phenotypic deformities and mortality rate in cotton mealybugs after silencing the *DNMT* genes by injection and topical delivery of siRNA. Each replicate consisted of 30 individuals that were analyzed at three-time points (24 h, 48 h, and 72 h) post-treatment. (**B**) The number of deformities corresponds to wing deformities. (**D**) The number of deformities corresponds to both black body color and dead offspring. Mortality and the number of deformities were normalized as mean ± SE with three biological replicates and analyzed by one-way ANOVA in conjunction with TUKEY test. Bars with different lowercase letters (abc) indicate significant differences between treatments (*p* < 0.05).

**Figure 5:**
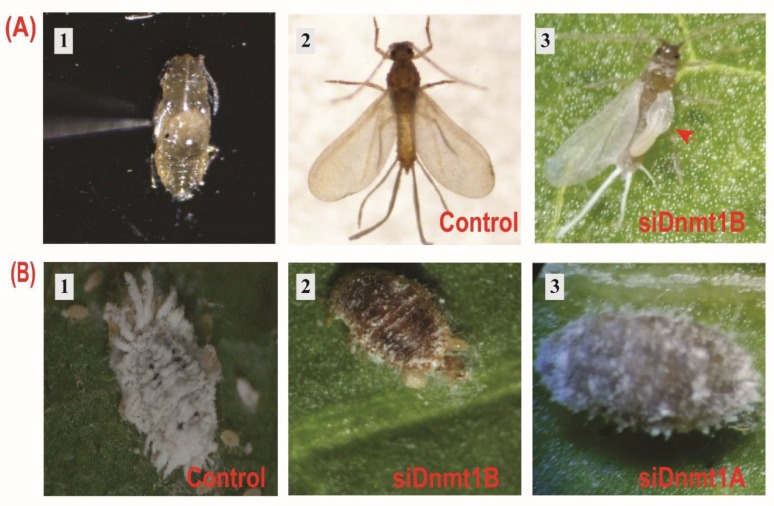
Abnormal phenotypes of survivors treated with siDnmt1A and 1B siRNAs. (**A**1) Newly molted pupa undergoing topical delivery with small interfering RNAs (siRNAs). (**A**2) Newly emerged adult male after 72 h post-treatment with a negative control with a single pair of well-developed wings. (**A**3) Newly emerged adult male after 72 h post-treatment with siDnmt1B with deformation wings. (**B**) Mortality in the offspring and abnormal color in adult females occurred in the adult females injected with siDnmt1A and 1B compared with the control. (**B**1) Adult gravid female with normal phenotype after injection with negative control. (**B**2,3) Adult gravid female with abnormal phenotype and dead offspring after 72 h post-treatment with siDnmt1B and siDnmt1A, respectively.

To study the functions of *PsDnmt1A* and *PsDnmt1B*, we used two methods to knock down the expression of these two genes in vivo. The first was to inject siRNAs into adult females and then observe the phenotypic changes in their offspring. The second was topical delivery of siRNAs to the pupae at the developmental stage with the highest expression level. A randomly shuffled siRNA was used as a negative control. All siRNA sequences were designed and confirmed to not share any sequence similarities with other cotton mealybug genes (Table 1). Both *PsDnmt1A* and *PsDnmt1B* were successfully silenced after siRNA treatment using the two methods (*t*-test, *p* < 0.05, Figure 3). Compared with the control groups, the *PsDnmt1A* expression level was significantly reduced in the pupa and adult females 24 h after siRNA treatment (*t*-test, *p* < 0.05, Figure 3A,C). The *PsDnmt1B*-specific mRNA level was significantly reduced after 72 h in the pupa stage (*t*-test, *p* < 0.05, Figure 3B), and it was significantly down-regulated after 24 h in adult females (*t*-test, *p* < 0.05, Figure 3D). After silencing *PsDnmt1A* and *PsDnmt1B* by injecting siRNAs into adult females, the treated adult females lost their white wax, and their body color became black 72 h post-treatment (Figure 4D and Figure 5B2,3). In addition, there were fewer offspring in the siRNA-treated group compared with the negative control (*t*-test, *p* < 0.05 Figure 4D and Figure 5B2,3). Furthermore, after knocking down *PsDnmt1B* using topical delivery in the pupal stages, we observed abnormal wing development in the adult males as well as the high mortality rate (*t*-test, *p* < 0.05, Figure 5A3).

## 4. Discussion

The cotton mealybug. *Phenacoccus solenopsis*, is one of the most destructive sap-sucking insects, and it has become an aggressive invasive pest in recent years. *P*. *solenopsis* infestations have caused huge economic crop losses in many countries around the world, including China [39,40,41]. Although DNMT enzymes actively methylate DNA at CpG sites, the mechanisms of de novo methylation and the maintenance function of DNMTs in insects are not well understood, especially because many insects lack DNMT3 [42]. We found that the cotton mealybug also lacks the *DNMT3* gene, similar to the red flour beetle, *T. castaneum*, and other insect species from the orders Lepidoptera, Hemiptera, and Hymenoptera [18,19,20,24,43]. However, there are two copies of DNMT1 present in the genomes of some insect species, such as the cotton mealybug, and other insect taxa, indicating that DNMT1 could assume the functions de novo methylation and also maintenance DNA methylation [24,44,45].

The *PsDnmt1B* gene was found to be the most highly expressed in gravid females (*t*-test, *p* < 0.05, Figure 2C), inferring that de novo methylation, if it does occur during embryonic development, might be catalyzed by *PsDnmt1B,* which has been proposed previously [46]. The *Dnmt1* gene in the brown planthopper *Nilaparvata lugens*, a hemipteran species, also exhibits similar expression profiles [10]. Furthermore, the sex-bias expression difference between males and females of *P*. *solenopsis* indicates that the *DNTM* genes could have a different function in regulating sexual dimorphism in the cotton mealybug. This is consistent with a previous study showing that the *DNMT* genes have different expression profiles in males and females of the sexually dimorphic Chinese white wax scale, *Ericerus pela* [47]. 

RNA interference is a powerful tool used to investigate gene functions [48]. However, RNAi is still challenging to use in the cotton mealybug because this insect has a life cycle that is very different from that of many other insects. *P. solenopsis* adult females are wingless and are covered with white wax, whereas the adult males do not have a wax covering, have wings, and are much smaller in size. Therefore, we tried two different RNAi methods to deliver siRNA into the cotton mealybugs. RNAi delivery methods have been documented in various insects, including hemipteran species [29,37,49,50,51,52]. The microinjection method is the most common method for RNAi delivery in scientific research and is time-consuming and requires a high degree of technical skill. However, one of the main advantages of this method is that it can deliver the RNAi directly and quickly into the insect hemolymph by avoiding possible barriers like the epithelium of the midgut [53]. Another method is topical delivery, which is suitable for small insects such as mealybug pupae in the present study; the pupae are not covered with white wax, unlike adult females which are heavily covered with wax that can prevent the absorbance of siRNA. Recent studies have shown that topical application of RNAi showed good delivery in treated aphids compared with other methods [37]. Our work shows that both methods are effective at knocking down genes in the cotton mealybug and that different methods need to be used to treat males vs. females. 

Our results show that silencing the *PsDnmt1A* gene in gravid females resulted in abnormal body color after losing their white wax form their body as well as offspring lethality compared with the control, and we observed a similar phenotype when we knocked down *PsDnmt1B.* However, knocking down expression of *PsDnmt1A* in pupae did not result in any abnormal phenotypes, while knockdown of *PsDnmt1B* in pupae led to failure of wing development in the adult stages. This observation may indicate that *PsDnmt1* is important during embryonic and tissue development in the cotton mealybug [54,55,56]. In the silkworm, *Bombyx mori*, the *BmDnmt1* gene is essential to ensure wing development by regulating tissue- and stage-specific transcription of *BmCHSA-2b* [57].

## 5. Conclusions

In summary, two paralogous copies of *DNMT1* were identified in the cotton mealybug genome. Our results show that *PsDnmt1A* and *PsDnmt1B* have different expression patterns in male and female mealybugs, indicating that these genes play different roles during male and female development. *PsDnmt1B* expression is more critical in the male development than is *PsDnmt1A*. Our results highlight, for the first time, the critical roles of DNA methylation in the cotton mealybug, *Phenacoccus solenopsis*. However, the full pathway or mechanism of DNA methylation, as well as the possible roles of the DNMT1 proteins and their effects on gene expression in the cotton mealybug, will require further study.

## Figures and Tables

**Table 1:** All primer pairs used in the present study.

Primer Name	Sense (5′–3′)	Antisense (5′–3′)	Purpose
DNMT1A	AGGACATCTGTGCCCATTC	CGACCATAAGTTGAGTCGTATCT	PCR
DNMT1A (1)	ATGAAGCAGGACATCTGTGCCCATTCG	GGAACGCCTCCTGTTGCATCGGA	RACE “1st round”
DNMT1A (2)	ATGCAACAGGAGGCGTTCCTATTAGACG	CCTTTTACGGCGATTGGTTCTCCAATCC	RACE “2nd round”
qDT1A	TACGCTGCTGGTTACGTTAAA	ACCGCTTCTTCACCTTCATC	qRT-PCR
DNMT1A-phso-490	GCCAGAGAAUUUAAAGGAUTT	AUCCUUUAAAUUCUCUGGCTT	siRNA synthesis
DNMT1A-phso-1542	CCAAGAAUUGACGUCGAUUTT	AAUCGACGUCAAUUCUUGGTT	siRNA synthesis
DNMT1A-phso-2093	GCUAGAUACGACUCAACUUTT	AAGUUGAGUCGUAUCUAGCTT	siRNA synthesis
DNMT1B	GAAGGTTTGCATCAAGCGGG	CCCATTGGTTCTGGTTGGGT	PCR
DNMT1B (1)	TCAGAGCAGGCGAAGGGCAATCA	TTGGCACGGAGGGCCACCGCACA	RACE “1st round”
DNMT1B (2)	GCATCAAGCGGGAATAGCGGAGTG	GCAAACCTTCTGTCAATCCTCCGCAAC	RACE “2nd round”
qDT1B	GGTCATCATCTGCTCCGTTAC	GGGATCGTGCTTATGAGGTATTT	qRT-PCR
DNMT1B-phso-1376	GCCAAGGAUUCAGUGGGAUTT	AUCCCACUGAAUCCUUGGCTT	siRNA synthesis
DNMT1B-phso-1771	GCUCCGUUACGGAUGCUAATT	UUAGCAUCCGUAACGGAGCTT	siRNA synthesis
DNMT1B-phso-2206	GCUAAUCGGCAUGGUAAUUTT	AAUUACCAUGCCGAUUAGCTT	siRNA synthesis
Negative control	UUCUCCGAACGUGUCACGUTT	ACGUGACACGUUCGGAGAATT	siRNA synthesis
PsActin	TCGTACCACCGGTATCGTATTA	TTAAGTCACGACCAGCCAAG	qRT-PCR

RACE, rapid amplification of cDNA ends; siRNA, small interfering RNA; qRT-PCR, quantitative reverse transcriptase-polymerase chain reaction.

## Supplementary Materials

The following are available online at https://www.mdpi.com/2075-4450/11/2/121/s1, Figure S1: The domain architecture of DNA (cytosine-5)-methyltransferase genes from *P. solenopsis* and the domain structure analysis was carried out by searching the Pfam database and then checked with HMMER and SMART., Table S1: Characteristics of DNA (cytosine-5)-methyltransferase genes of the cotton mealybug *Phenacoccus solenopsis*, Table S2: Homologs of sequences for phylogenetic trees, Table S3: Full sequences of DNMT1 genes of cotton mealybug *Phenacoccus solenopsis*.
