# Supplementary material for: The Roles of DNA Methyltransferases 1 (DNMT1) in Regulating Sexual Dimorphism in the Cotton Mealybug, Phenacoccus solenopsis
Source: Insects. 2020 Feb 12;11(2):121. doi: 10.3390/insects11020121 (PMC7074402; doi:10.3390/insects11020121)
Supplement: Supplementary file 1 [file insects-11-00121-s001.pdf]

The roles of DNA methyltransferases (DNMTs) in regulating sexual dimorphism in the cotton mealybug, *Phenacoccus solenopsis*

Mohamed A.A. Omar [1,2], Meizhen Li [1], Feiling Liu [1], Kang He [1], Muhammad Qasim [1], Huamei Xiao [1,3], Mingxing Jiang [1], and Fei Li [1,*]

1. Ministry of Agriculture Key Lab of Molecular Biology of Crop Pathogens and Insects / Institute of Insect Science, Zhejiang University, 866 Yuhangtang Road, Hangzhou 310058, China
2. Department of Plant Protection, Faculty of Agriculture (Saba Basha), Alexandria University, Alexandria, Egypt
3. College of Life Sciences and Resource Environment / Key Laboratory of Crop Growth and Development Regulation, Jiangxi Province, Yichun University, Yichun 336000, China

*** Corresponding author: Dr. Fei Li, Email: lifei18@zju.edu.cn**

Supporting Information

Table S1: Characteristics of DNA (cytosine-5)-methyltransferase genes of the cotton mealybug *Phenacoccus solenopsis*

Gene	cDNA length	Coding region	5'UTR	3UTR	aa	pI	Mw (Da)	Accession number
PsDnmt1A	2225	231-2123	230	101	630	5.88	73111	MN696786
PsDnmt1B	2862	>3-2069	>3	392	822	8.25	80030	MN696787

Abbreviation: aa, amino acid; Mw, molecular weight; pI, isoelectric point; UTR, untranslated region.

Table S2: Homologs of sequences for phylogenetic trees

DNMTs	Accession number	Species	Order
BgDnmt1	PSN43414.1	*Blattella germanica*	Blattodea
CsDnmt1	PNF30164.1	*Cryptotermes secundus*	Blattodea
TcDnmt1_isoform2	ASA69506.1	*Tribolium castaneum*	Coleoptera
TcDnmt1_isoform1	XP_008193458.1	*Tribolium castaneum*	Coleoptera
ApDnmt1	XP_025835356.1	*Agrilus planipennis*	Coleoptera
OtDnmt1	XP_022900238.1	*Onthophagus taurus*	Coleoptera
TcDnmt2	XP_972690.1	*Tribolium castaneum*	Coleoptera
ApDnmt2	XP_018332335.1	*Agrilus planipennis*	Coleoptera
LdDnmt2	XP_023025667.1	*Leptinotarsa decemlineat*	Coleoptera
OtDnmt2	XP_022909983.1	*Onthophagus taurus*	Coleoptera
DmDnmt2	AAF03835.1	*Drosophila melanogaster*	Diptera
NlDnmt1	AHZ08393.1	*Nilaparvata lugens*	Hemiptera
NlDnmt1a	XP_022199001.1	*Nilaparvata lugens*	Hemiptera
MpDnmt1	XP_022173545.1	*Myzus persicae*	Hemiptera
MsDnmt1	XP_025199418.1	*Melanaphis sacchari*	Hemiptera
PsDnmt1A	MN696786	*Phenacoccus solenopsis*	Hemiptera
PsDnmt1B	MN696787	*Phenacoccus solenopsis*	Hemiptera
NlDnmt2	XP_022192816.1	*Nilaparvata lugens*	Hemiptera
MpDnmt2	XP_022162096.1	*Myzus persicae*	Hemiptera
NlDnmt3	ALA15698.1	*Nilaparvata lugens*	Hemiptera
BtDnmt3b	ATN96644.2	*Bemisia tabaci*	Hemiptera
PsDnmt2	PSOL09183	*Phenacoccus solenopsis*	Hemiptera
MsDnmt2	XP_025201508.1	*Melanaphis sacchari*	Hemiptera
BtDnmt3_isoform_X1	XP_018899088.1	*Bemisia tabaci*	Hemiptera
TCDnmt2	XP_024888713.1	*Temnothorax curvispinosus*	Hyemnoptera
HlDnmt1	XP_017792154.1	*Habropoda laboriosa*	Hyemnoptera

BiDnmt1_isoform_X2	XP_012246575.1	*Bombus impatiens*	Hyemnoptera
ACDnmt1	PBC25685.1	*Apis cerana cerana*	Hyemnoptera
MqDnmt1	KOX74441.1	*Melipona quadrifasciata*	Hyemnoptera
BtDnmt1	XP_020723291.1	*Bombus terrestris*	Hyemnoptera
TzDnmt1	XP_018308579.1	*Trachymyrmex zeteki*	Hyemnoptera
OaDnmt1	XP_023289399.1	*Orussus abietinus*	Hyemnoptera
NvDnmt1	NP_001164521.1	*Nasonia vitripennis*	Hyemnoptera
BiDnmt1b	XP_003489082.1	*Bombus impatiens*	Hyemnoptera
HsDnmt1a	XP_011151720.1	*Harpegnathos saltator*	Hyemnoptera
AdDnmt1a	XP_006612376.1	*Apis dorsata*	Hyemnoptera
AfDnmt1a	XP_012340685.1	*Apis florea*	Hyemnoptera
AdDnmt.1a	XP_006612375.1	*Apis dorsata*	Hyemnoptera
AcDnmt1a	XP_012060955.1	*Atta cephalotes*	Hyemnoptera
WcDnmt1a	XP_011704997.1	*Wasmannia auropunctata*	Hyemnoptera
TCDnmt2	XP_024888713.1	*Temnothorax curvispinosus*	Hyemnoptera
DqDnmt1b	XP_014470488.1	*Dinoponera quadriceps*	Hyemnoptera
AfDnmt1b	XP_012339009.1	*Apis florea*	Hyemnoptera
MdDnmt1b	XP_008547166.1	*Microplitis demolitor*	Hyemnoptera
PcDnmt1b	XP_014605619.1	*Polistes canadensis*	Hyemnoptera
BiDnmt1a	XP_012246574.1	*Bombus impatiens*	Hyemnoptera
CfDnmt1a	XP_011257755.1	*Camponotus floridanus*	Hyemnoptera
Sidnmt2	XP_011158525.1	*Solenopsis invicta*	Hyemnoptera
HiDnmt2	XP_017795539.1	*Habropoda laboriosa*	Hyemnoptera
AdDNMT3_isoform_X1	XP_006618258.1	*Apis mellifera*	Hyemnoptera
AmDnmt3_isoform_X1	XP_026302146.1	*Apis mellifera*	Hyemnoptera
CcDnmt2	XP_017888112.1	*Ceratina calcarata*	Hyemnoptera
AmDnmt2	XP_006563008.1	*Apis mellifera*	Hyemnoptera
ArDnmt2	XP_012261891.1	*Athalia rosae*	Hyemnoptera
BiDnmt2	XP_012249327.1	*Bombus impatiens*	Hyemnoptera

MqDnmt2	KOX67520.1	*Melipona quadrifasciata*	Hyemnoptera
AmDNMT3	XP_026302147.1	*Apis mellifera*	Hyemnoptera
AmDnmt3_isoform_X3	XP_026302149.1	*Apis mellifera*	Hyemnoptera
AmDnmt3_isoform_X4	XP_026302149.1	*Apis mellifera*	Hyemnoptera
BtDnmt3	XP_020723291.1	*Bombus terrestris*	Hyemnoptera
BtDnmt3a	NP_001340321.1	*Bombus terrestris*	Hyemnoptera
AcDnmt3	AFK33206.1	*Apis cerana cerana*	Hyemnoptera
AmDnmt3_isoform_X2	XP_026302147.1	*Apis mellifera*	Hyemnoptera
AmDnmt3_isoform_X5	XP_026302150.1	*Apis mellifera*	Hyemnoptera
CcDnmt1	XP_017882042.1	*Ceratina calcarata*	Hymenoptera
CcDnmt1_isoform_X1	XP_015604098.1	*Cephus cinctus*	Hymenoptera
AmDnmt1a	NP_001164522.2	*Apis mellifer*	Hymenoptera
SiDnmt1_isoform_X3	XP_025996916.1	*Solenopsis invicta*	Hymenoptera
SiDnmt1_isoform_X2	XP_025996917.1	*Solenopsis invicta*	Hymenoptera
ArDnmt1_isoform_X2	XP_020707405.1	*Athalia rosae*	Hymenoptera
ArDNMT1_isoform_X3	XP_012254091.1	*Athalia rosae*	Hymenoptera
ArDNMT1_isoform_X1	XP_012254091.1	*Athalia rosae*	Hymenoptera
CfDnmt1_isoform_X1	XP_025263172.1	*Camponotus floridanus*	Hymenoptera
DnDnmt1	XP_015431301.1	*Dufourea novaeangliae*	Hymenoptera
TpDnmt1	XP_014233345.1	*Trichogramma pretiosum*	Hymenoptera
AeDnmt1a	XP_011056477.1	*Acromyrmex echinatior*	Hymenoptera
PbDnmt1	XP_011631933.1	*Pogonomyrmex barbatus*	Hymenoptera
Obdnmt1_isform_x2	XP_011350635.1	*Ooceraea biroi*	Hymenoptera
AcDnmt1	KYM80462.1	*Atta colombica*	Hymenoptera
PbDnmt2	XP_011635367.1	*Pogonomyrmex barbatus*	Hymenoptera
ObDnmt2	XP_011351845.1	*Ooceraea biroi*	Hymenoptera
AcDnmt2	XP_018043909.1	*Atta colombica*	Hymenoptera
AeDnmt2	XP_011062299.1	*Acromyrmex echinatior*	Hymenoptera
ZnDnmt1	XP_021941799.1	*Zootermopsis nevadensis*	Isoptera

ZnDnmt2	XP_021928076.1	*Zootermopsis nevadensis*	Isoptera
SlDnmt2	XP_022819860.1	*Spodoptera litura*	Lepidoptera
PmDnmt1	KPJ15240.1	*Papilio machaon*	Lepidoptera
TnDnmt1	XP_026734338.1	*Trichoplusia ni*	Lepidoptera
PrDnmt1	XP_022127650.1	*Pieris rapae*	Lepidoptera
PxDnmt1	KPI97703.1	*Papilio xuthus*	Lepidoptera
PxDnmt	AWI66421.1	*Plutella xylostella*	Lepidoptera
BmDnmt1	BAP86925.1	*Bombyx mori*	Lepidoptera
BmDnmt1_isoform_X1	XP_012550860.1	*Bombyx mori*	Lepidoptera
HaDnmt1	XP_021188281.1	*Helicoverpa armigera*	Lepidoptera
DpDnmt1	OWR52605.1	*Danaus plexippus plexippus*	Lepidoptera
VtDnmt1	XP_026492162.1	*Vanessa tameamea*	Lepidoptera
SlDnmt2	XP_022819860.1	*Spodoptera litura*	Lepidoptera
PrDnmt2	XP_022114322.1	*Pieris rapae*	Lepidoptera
PxDnmt2	XP_013182102.1	*Papilio xuthus*	Lepidoptera
HaDnmt2	XP_021181237.1	*Helicoverpa armigera*	Lepidoptera
BmDnmt2	XP_021202737.1	*Bombyx mori*	Lepidoptera
FoDnmt1	XP_026282587.1	*Frankliniella occidentalis*	Thysanoptera
FoDnmt2	XP_026277115.1	*Frankliniella occidentalis*	Thysanoptera

Table S3: Full sequences of DNMT1 genes of cotton mealybug *Phenacoccus solenopsis*

Gene name	Full sequences
PsDnmt1A	mRNA sequence:<br><br>ACATGGGGTACAACAAGAGGGTTCCAGCTACCAGTTGATTTTATACGTTATATTTTGAAACACATTGCGAATTCCGTTAGTGGCGGTTGCGAATTGTAAATTTGTGAAATGAGTTTGATTTCCACTGTCATCATGGAAT<br>AATATTTTATTGTTCAGTCAAATTAAAACTAATTCATTTTCGATTTATTCAGACTTTTTTTCCACCTTGGTAACACGGCGTGCAGAATAATATGGATGATGAACGTTTGAGAAACACATTGAATTATGAACGAAGAAAA<br>CTGAAAGAGCAATTCATACGCGAAAATTTGGAAAGAGTTGAAAATATAAGAAATCCAAATAAACGAAAAATAACCGAAGAACTATCAAACTATGACAAACGATCAAGAACAGACAACTATGATGTTTCAGACGAA<br>TCTAACGAAAATATACCCCTAAAGAGGAAAAGCAACTTATTCCGAAGTTGTGCGCCAGAGAATTTAAAGGACGAATATCCATATGAAATTGATGATCGGAAAAAAAGGACTTCTATTTGCGATTTTTGTCGTCAATC<br>GTTGAAGGTAGCCGATGAATCTCGATTTGATAGCAGTGATTTTGTGGCATCTAAAAAAGAAAGTTCCATTTTATTCAACAATAAATTGAATGTTTTTCCCGAAGATGACAAACTAGGAGTAAAATTAACAGCTTTCAG<br>CGTATACGATGAAGCAGGACATCTGTGCCCATTCGACAATGATTATATTGAAGACGGGGTCGTTTTATACGCTGCTGGTTACGTTAAAGCTATACATAGTGAAAAATCCGATGCAACAGGAGGCGTTCGTATTAGAC<br>GAATTGGTCCTATTTCAGCATGGTGGATAACTGGTTTCGATGAAGGTGAAGAAGCGGTGTTAGGGATATCAACAGAAATCGCTGATTATTATCTGATGAAAGCGAGCGAAAAATATCAACCTTTTCTGAAAGATATG<br>CATCAAAAAATGTACTTAGTCAAGATAATAGTTGAATTTTTACAAAAAAGCGAAGAAGGCGAAGCTACATACGAAAATTTACTGTACAAATTGAAGAATACTGTTCCTCCTTCTGGATTTAAATCGCTTTCAGAAGA<br>AACTTTACTGCCTTACGCCAATTACATTTACGATCTTGTTTTAAGCTACGATTCGTCCGCAGATTCTGCAGAACGATTGCTCGTTAATACACCGTGTATGAAACATTTAGCCAAACTAGCGGGAATTTCCGCTGAAAAA<br>AGACTCGAGAAAATACGTCGAAAGAAGAAAACGAGTTGGATTAAAAGAAATAACGAAATGTCCAAAGTTGCTACTACAAATCTAGTAGAAAATATTTTTGAATCCTTTTTTTCTGAACAAATGGATAAGAGTTGTAA<br>CGATTCAATTATGTTGAATGAATGTTTTGTATTGTTACATAAATGTGAGTTCCGCGATGAGAACGAAAATAAAATCGCTGATGAAGAAAATACGAGCAGTGCCACGAGCAAGTTGAAAAATTCTTCATTTCAGACTC<br>GAGAGTGTTCAGTTGTATTGCCAAGAATTGACGTCGATTCATATTATAAATGCGAGGAAAAATCGCAAATGACTTGTAATCTCGTAAAGCCGTTTTCAATTGTGCTGGAAACGTTGTCTGATGAAATAATCGAAAAAT<br>ATTCACGCAAAGGCCAGGATGCTGGAAATAATGCAGTATCGAGGAGGAAGAAACGATATATCCGAAACAGCAGTTATTCCAACATCATTGTAAAATGGATTGGAGAACCAATCGCCGTAAAAGGAAATAGAAGTTA<br>TTATCGATGTGCTGCAATTTCGTCTTTGGAAATTATGGTCAACGATTTCGTTACTGTCAACGATGAAAACCCAAGAACGGATGATCGTATCGGCCGTATCGCTTATATGTGGGAAGAAAATAATAAGCAAAAATTCCA<br>TATCAACTGGATGATAAAAAGCGTGAAAACAATTCTTGGTGATACTGGAAACCCTAAAGAATTATTCGAAACTTATGACTGTGATAATTTATTCTTGAGTGAGGTAAACGACAGAGTAAAAGTGACGTACCTTCCTA<br>TACCCAATAAAAATTGGATCACGCTAGATACGACTCAACTTATGGTCGATTTGAAGACATAGCACCGTGTATGAAACATTTAGCCAAACTAGCGGGAATTTCCGCTGAAAAAAGACTCGAGAAAATACGTCGAAAA<br>AAGAAAAAAAAAAAAAAAAAAAAAAAA<br><br>Protein sequence:<br><br>MDDERLRNTLNYERRKLKEQFIRENLERVENIRNPNKRKITEELSNYDKRSRTDNYDVSDESNENIPLKRKSNLFRSCAPENLKDEYPYEIDDRKKRTSICDFCRQSLKVADESRFDSSDFVASKKESSILFNNKLNVFPEDDKLG<br>VKLTAFSVYDEAGHLCPFDNDYIEDGVVLYAAGYVKAIHSEKSDATGGVRIRRIGPISAWWITGFDEGEEAVLGISTEIADYYLMKASEKYQPFLKDMHQKMYLVKIIVEFLQKSEEGEATYENLLYKLKNTVPPSGFKSLSEE<br>TLLPYANYIYDLVLSYDSSADSAERLLVNTPCMKHLAKLAGISAEKRLEKIRRKKKTSWIKRNNEMSKVATTNLVENIFESFFSEQMDKSCNDSIMLNECFVLLHKCEFRDENENKIADEENTSSATSKLKNSSFQTRECSVVLP<br>RIDVDSYYKCEEKSQMTCNLVKPFSIVLETLSDEIIEKYSRKGQDAGNNAVSRRKKRYIRNSSYSNIIVKWIGEPIAVKGNRSYYRCAAISSLEIMVNDFVTVNDENPRTDDRIGRIAYMWEENNKQKFHINWMIKSVKTILGDT<br>GNPKELFETYDCDNLFLSEVNDRVKVTYLPIPNKNWITLDTTQLMVDLKT
PsDnmt1B	mRNA sequence:<br><br>ACATGGGCAACGATGAAAACCCAAGAACGGATGATCGTATCGGCCGTATCGCTTATATGTGGGAAGAAAATAATAAGCAAAAATTCCATATCAACTGGATGATAAAAAGCGTGAAAACAATTCTTGGTGATACTGG<br>AAACCCTAAAGAATTATTCGAAACTTATGACTGTGATAATTTATTCTTGAGTGAGGTAAACGACAGAGTAAAAGTGACGTACCTTCCTATACCCAATAAAAATTGGATCACGCTAGGTAATTTCTTCTTAAATGAATG<br>GAATAGTTCAGCTAATTCGAAAGATGAAAAATCGTTTTACTTTCAAAAAAGATACGACTCAACTTATGGTCGGTTTGAAGACATAGAAATTGGTATATCTGAAAATAAAAATTTCGATATGTGTCCACGTTGCGAGC<br>ATATGAAAATGAAGAAGAATTGGGAGAAACCTTGTCTTGTCGAAGAACTCGAATATATTGAAAGCACCGATGAATACTCTTACGGTATGGTACGCTGGAGAGATGAAGAACTGAAAATAGGTAGCTGCGTATTTTTG<br>AAACCTGGTACACTTTTTCCAATACTGAATAAAAGTTATAACGAAAAGGTCAAGTTGAAAAAATTGGATGAAGATCGCTATCCCGAAATGTATAGAAAACAGATAGATCGCACTAAGTTCGAAGAAGTTGCAGAAC<br>CATTCAATATCGGTTACATTGTATCGATACATTCAGAAAAAAGGTGTAAATCGGCCTCTGATATATTTTTGACTGTTAAAAAATTTTACCGACCTCAAGATACTCATTTAGACGAGGATACTGTCAAACATCTTGACTT<br>AAATTTGCTATATTGGAGCGAAGAAGAATCAGTTGTAAGTTTCACGAAAGTAGAAGGAAAATGTTACGTGGCTAATGAAAAGAATTTGAAGTGTTCAACTGATGAGTGGAGTCAACAAGGACCTTATAGATTTTATT<br>ACAAAAATGCATACGATCATAAAAATAAGTCGTTCAGTCGTGCTCCAACAACAATAACGCGATATTATGAAGATCCTGTTACTCGCAGAAAATCCAATATTATTGAAGAAATAATTGAGGAGAATCCTGTTCTTTGG<br>CCTACCATATCAAGACCATTACGTTGTTTGGATATTTTCGCTGGTTGCGGAGGATTGACAGAAGGTTTGCATCAAGCGGGAATAGCGGAGTGTCGATGGGCAATTGAAAAAGAGGAAGCCGCAGCTCATTCTTTTCG<br>ACTGAATTATCCTGAAGCTTCTGTTTTCACCGAAGATTGCAATGAGCTTTTGAAATTTATGATGAATGGTAAATTCAAAAAAAAATGGTCAATATTTACCTCAGAAAGGTGAAGTCGAGTTATTGTGCGGTGGCCCTCC<br>GTGCCAAGGATTCAGTGGGATGAACCGTTTTAACCATCTTCAATACTCTAGTTTCAAGAATTCATTGGTCGTTTCTTTTCTGTCTTACGTCGATTTTTTCCGTCCTCGTTTTGTGATCATGGAAAATGTTAGAAATTTTGT<br>GCTGTTTAAAAAAAGCATAGTATTGAAACTTACCCTCAGATGTCTAATTAAAATGGGTTATCAGTGTACTTTCGGAATCGTTCAAGCCGGTAATTACGGAGTTCCTCAGAGCAGGCGAAGGGCAATCATTTTAGCGGC<br>AGCACCCGGCGAAGCTTTGCCGAATTTTCCGGAGCCCATGCACGTTTTTGTTAGTGGGGCAACCACTTTGAATGTTGATGATACAAAATTTATGACCAACACGAAATGGTCATCATCTGCTCCGTTACGGATGCTAAG<br>CGTTTATGATGCGATTAGTGATTTGCCCGAAATTAAAGCAGGTGATTTGTCACGAATCGAAATACCTCATAAGCACGATCCCAAAACTCACTTCCAAAGACTGATTAGAAAATCCAACAGTGGTGATGTATTGCTCAA<br>AGATCATGTCTGTAAACCGATGAACCTATTAGTGGAGAAGCGTATTTCCTTCATTCCTCGCGCACCTGGTTCCGATTGGAGAGATTTACCAAACATCGAAGTAAAACTTCGAAACGGAAGCATGATAAAAAAACTAA<br>TTTATACGCATCACGACATAAAAAGTGGTAAATCAAAACAAGGACGTTTACGTGGAGTTTGTTCTTGTGCTTCTGGTGGAAAATGTAAACCAAAGTACAAACAAAATGATACATTAATTCCATGGTGTCTTCCTCATA<br>CCGCTAATCGGCATGGTAATTGGTCTGGACTTTATGGACGTATTGAATGGGATAAATTTTTCAGAACAGTTGTTACCCAACCAGAACCAATGGGTAAACAGGGAAAAGTGATACATCCCGAAGAAAATCGTGTGATA<br>TCTGTTCGAGAAAGTGCGCGTTCCCAAGGTTTTCCGGACAGTTTTCAGTTTTTCGGAAGTATCTCTGATAAATACAGACAAATTGGTAATGCAGTTCCGCCTCCTTTGGCTCTTGCTTTGGGACTGGTCTAAAATCGGC<br>AGCTTATGCTGAGAAGTGAAAAAAGCTTTTAATATTTGGACGAATTTAGTTTTCTATTTTTTTTTAAATGGTTTTTCTATTATCGTTATGGAATTTGTTTTTTGATGTTTTCATTCATTACATGAGTTGTTGATTTTTTATC<br>ATTGTATTTTTTTTTTTGTTTATTTTTTAAAGTAAATGCCAATAAGGCTTTCAAGTGATGATTTAATATTCATTTCAATTGTAGTGGTCTAATTTCATTTTCAACTTATTATATATTTTAACGAAGTAGAATTTTTTCATCTTTC<br>CCTGCTACTTAATATAAGTTGATGTTTGTTTATTTACTGTGATATAAATAAAATTTGAGTGCGGCTGGTAAAAAAAAAAAAAAAAAAAAAAAAAAAAAAA<br><br>Protein sequence:<br><br>MGNDENPRTDDRIGRIAYMWEENNKQKFHINWMIKSVKTILGDTGNPKELFETYDCDNLFLSEVNDRVKVTYLPIPNKNWITLGNFFLNEWNSSANSKDEKSFYFQKRYDSTYGRFEDIEIGISENKNFDMCPRCEHMKMKK<br>NWEKPCLVEELEYIESTDEYSYGMVRWRDEELKIGSCVFLKPGTLFPILNKSYNEKVKLKKLDEDRYPEMYRKQIDRTKFEEVAEPFNIGYIVSIHSEKRCKSASDIFLTVKKFYRPQDTHLDEDTVKHLDLNLLYWSEEESVVS<br>FTKVEGKCYVANEKNLKCSTDEWSQQGPYRFYYKNAYDHKNKSFSRAPTTITRYYEDPVTRRKSNIIEEIIEENPVLWPTISRPLRCLDIFAGCGGLTEGLHQAGIAECRWAIEKEEAAAHSFRLNYPEASVFTEDCNELLKFMM<br>NGKFKKNGQYLPQKGEVELLCGGPPCQGFSGMNRFNHLQYSSFKNSLVVSFLSYVDFFRPRFVIMENVRNFVLFKKSIVLKLTLRCLIKMGYQCTFGIVQAGNYGVPQSRRRAIILAAAPGEALPNFPEPMHVFVSGATTLNVD<br>DTKFMTNTKWSSSAPLRMLSVYDAISDLPEIKAGDLSRIEIPHKHDPKTHFQRLIRKSNSGDVLLKDHVCKPMNLLVEKRISFIPRAPGSDWRDLPNIEVKLRNGSMIKKLIYTHHDIKSGKSKQGRLRGVCSCASGGKCKPKYK<br>QNDTLIPWCLPHTANRHGNWSGLYGRIEWDKFFRTVVTQPEPMGKQGKVIHPEENRVISVRESARSQGFPDSFQFFGSISDKYRQIGNAVPPPLALALGLV

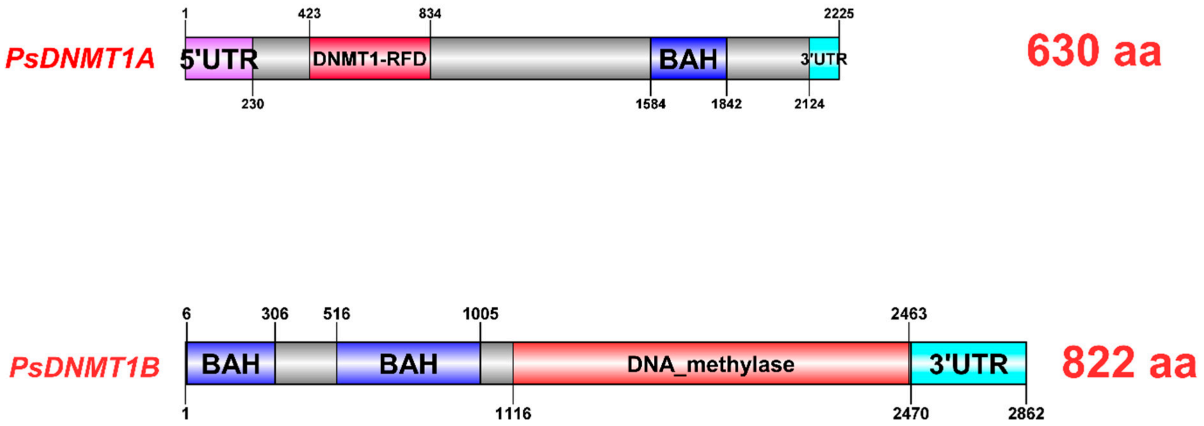

Figure S1: The domain architecture of DNA (cytosine-5)-methyltransferase genes from *P. solenopsis* and the domain structure analysis was carried out by searching the Pfam database and then checked with HMMER and SMART.
